# Rapid but specific perceptual learning partially explains individual differences in the recognition of challenging speech

**DOI:** 10.1038/s41598-022-14189-8

**Published:** 2022-06-15

**Authors:** Karen Banai, Hanin Karawani, Limor Lavie, Yizhar Lavner

**Affiliations:** 1 Department of Communication Sciences and Disorders, University of Haifa, Haifa, Israel; 2 Department of Computer Science, Tel-Hai College, Qiryat Shemona, Israel

**Keywords:** Psychology, Auditory system, Learning and memory, Sensory processing, Cognitive neuroscience, Language, Perception

## Abstract

Perceptual learning for speech, defined as long-lasting changes in speech recognition following exposure or practice occurs under many challenging listening conditions. However, this learning is also highly specific to the conditions in which it occurred, such that its function in adult speech recognition is not clear. We used a time-compressed speech task to assess learning following either brief exposure (rapid learning) or additional training (training-induced learning). Both types of learning were robust and long-lasting. Individual differences in rapid learning explained unique variance in recognizing natural-fast speech and speech-in-noise with no additional contribution for training-induced learning (Experiment 1). Rapid learning was stimulus specific (Experiment 2), as in previous studies on training-induced learning. We suggest that rapid learning is key for understanding the role of perceptual learning in online speech recognition whereas longer training could provide additional opportunities to consolidate and stabilize learning.

## Introduction

The recognition of connected speech (i.e., utterances longer than one word) under adverse conditions (e.g., distortion, background noise), which are abundant in daily listening environments, can be challenging^1^, but practice can lead to substantial improvements^2–9^. These improvements reflect perceptual learning, defined as relatively long-lasting changes in the ability to extract information from the environment following experience or practice^10–12^. However, stimulus specificity is considered a hallmark of perceptual learning^11,13–15^, and perceptual learning of speech is indeed often specific to the acoustic characteristics of the stimuli encountered during practice^16–19^. On the other hand, many sources of acoustic variability are present in typical listening environments (e.g., different talkers, different types and levels of background noise etc.)^1,20^, making it unlikely that acoustically-specific past learning could support ‘real-world’ future speech recognition. Alternatively, rapid learning could support online perception in challenging conditions by allowing listeners to quickly adapt to the acoustic characteristics of a broad range of conditions^21,22^. Consistent with this hypothesis, recent studies suggest that perceptual learning ‘clusters’ across a range of conditions and could thus form an individual capacity^23–26^. Furthermore, we found that rapid improvements in the recognition of one type of acoustically challenging speech are associated with individual differences in the recognition of other forms of challenging speech^22,27,28^. Nevertheless, as we focused on rapid changes, it is not clear whether this learning reflects perceptual learning as defined above (that is: relatively long-lasting). Therefore, in the current study we ask whether rapid perceptual learning that is retained over time is associated with individual differences in speech recognition (Experiment 1), and whether this learning is as stimulus specific as found in previous training studies^17,29^ (Experiment 2).

In the context of speech, perceptual learning has been studied for both speech-parts (e.g., syllables, phonemic categories) and connected speech (e.g., sentence recognition)^12^. Whereas the former clearly reflects perceptual learning, it could be argued that improvements in the recognition of connected speech under adverse conditions reflect higher-level linguistic or attentional processes, especially when it occurs rapidly. However, models of perceptual learning explicitly acknowledge the role of these processes. For example, according to the Reverse Hierarchy Theory (RHT^30^) rapid perceptual learning operates on high-level representations of speech, that are behaviorally relevant in a speech recognition scenario. Others^31^ argued that the key to perceptual learning is sufficient stimulus-related neural activity. In connected speech recognition, linguistic context can enhance this activity and therefore support perceptual learning^1,4^. Our definition of perceptual learning is neutral with respect to the processes modified by learning. Instead, we focus on the longevity and stimulus specificity of rapid learning. There is some indication that even rapid learning of speech is maintained over time^32,33^. In contrast, results for the specificity of either rapid or training-induced perceptual learning of connected speech are mixed^5,6,16,18,28,34–40^. For time-compressed speech, some studies found that learning was not specific to the compression rate and even transferred from time-compressed to natural-fast speech^5,37^, but others did not^28,29,41^. Methodological differences make the outcomes hard to compare across studies. Therefore, one goal of the current study was to test the talker specificity of rapid learning of time-compressed speech across different rapid learning protocols and test times (immediate and delayed).

### The potential role of perceptual learning in speech recognition

Theories of both perceptual learning^30^ and speech processing^42,43^ suggest that encounters with speech input trigger implicit and largely automatic processes which attempt to match this input to long-held representations. However, in daily listening situations automatic matching can fail due to lack of agreement between new inputs and long-term representations (e.g., due to sources of acoustic variability like noise or accent. Theoretically, this failure can trigger a learning process that gradually allows listeners to resolve finer-grained acoustic details and help them recognize previously unrecognizable input^30^. Because learning is triggered by a specific input, learning is at least partially specific to the acoustics of the input^7,30,42^. This specificity probably constrains the role of learning in complex communication environments. One option is that intensive experience is required to yield learning that supports speech recognition. However, training-induced learning of challenging speech is often quite specific to the trained stimuli^18,40,44,45^. Therefore, it can support future speech perception only to the extent that newly encountered situations replicate the conditions encountered in training. Therefore, intensive training studies are not a good analogue for real life conditions when a practice period is unlikely and the acoustics can change rapidly (e.g., in a multi-talker conversation). Consistent with this view, training often fails to yield quantifiable benefits in any untrained conditions, despite good learning on the trained ones even in listeners with perceptual difficulties (e.g., due to hearing loss)^19,46,47^. Studies on learning new speech categories [e.g.,^13^, ^33^] are also not a good approximation for daily environments because they usually do not use connected speech.

Another potential role of perceptual learning which we pursue here is based on rapid learning: if learning occurs rapidly, it could serve as a skill listeners can recruit whenever they encounter new acoustic challenges. Accordingly, specific learning could afford optimal adaptation to the particulars of a new acoustic challenge without more general and undesirable changes in speech perception. Rapid-learning studies are more representative of real-world challenges than training studies because they often include little stimulus repetition and connected speech materials^4,5,28,34,35,48,49^. Therefore, this account is more ecological than accounts based on the generalization of past learning. Consistent with the idea that perceptual learning is a general resource, recent findings show that learning is correlated across different tasks and even across modalities^23,24,26^.

## Overview of the current study

We conducted two experiments using time-compressed speech to elicit learning. In Experiment 1, we compared learning and retention between rapid and training-induced learning of time-compressed speech, to determine whether rapid learning conforms to the definition of perceptual learning. We also asked whether the two types of learning are differentially correlated with speech recognition in two different tasks—speech-in-noise and natural-fast speech. We report that rapid learning was maintained over time, consistent with the definition of perceptual learning. Furthermore, perceptual learning of time-compressed speech was associated with the perception of natural-fast speech and speech-in-noise, with no apparent differences between rapid and training-induced learning. Experiment 2 focused on the characteristics of rapid learning by exploring the effects of stimulus repetition and talker variability on rapid perceptual learning of time-compressed speech. Comparison of the outcomes to those of previous studies on learning following training^16,17,29^ suggests that rapid learning of time-compressed speech is as specific as training-induced learning.

## Experiment 1

### Methods

#### Participants

A total of 160 university students or recent graduates (ages 18–35 years, Mean = 26, SD = 3, 91 female and 69 male) participated in this experiment. Participants were volunteers and reported they were native Hebrew speakers, with normal hearing and no history of attention, learning or language deficits and no experience with time-compressed speech. The study was performed in accordance with the declaration of Helsinki. All aspects of the study were approved by the ethics committee of the Faculty of Social Welfare and Health Sciences at the University of Haifa (permit #199/12). Informed consent was obtained from all participants. Participants were tested as described below; no other tests were conducted.

Participants were divided randomly to two groups, a rapid-learning group that was exposed to time-compressed speech during testing but received no additional training and a training group that was tested like the other group and also completed additional training as described below. Both groups completed two test sessions on separate days, in which they performed the speech tasks described below. We note that parts of the data from the rapid-learning group were previously published as part of a conference proceedings^50^, and re-analyzed for the purpose of the current study. One participant had missing data and was not included in data analysis, so we report data from 79 listeners in the *rapid-learning* group (age: Mean = 26, SD = 4; 38 female, 41 male) and 80 listeners in the *training* group (ages: Mean = 26, SD = 3; 52 female, 28 male).

#### Overall design

**Figure 1:**
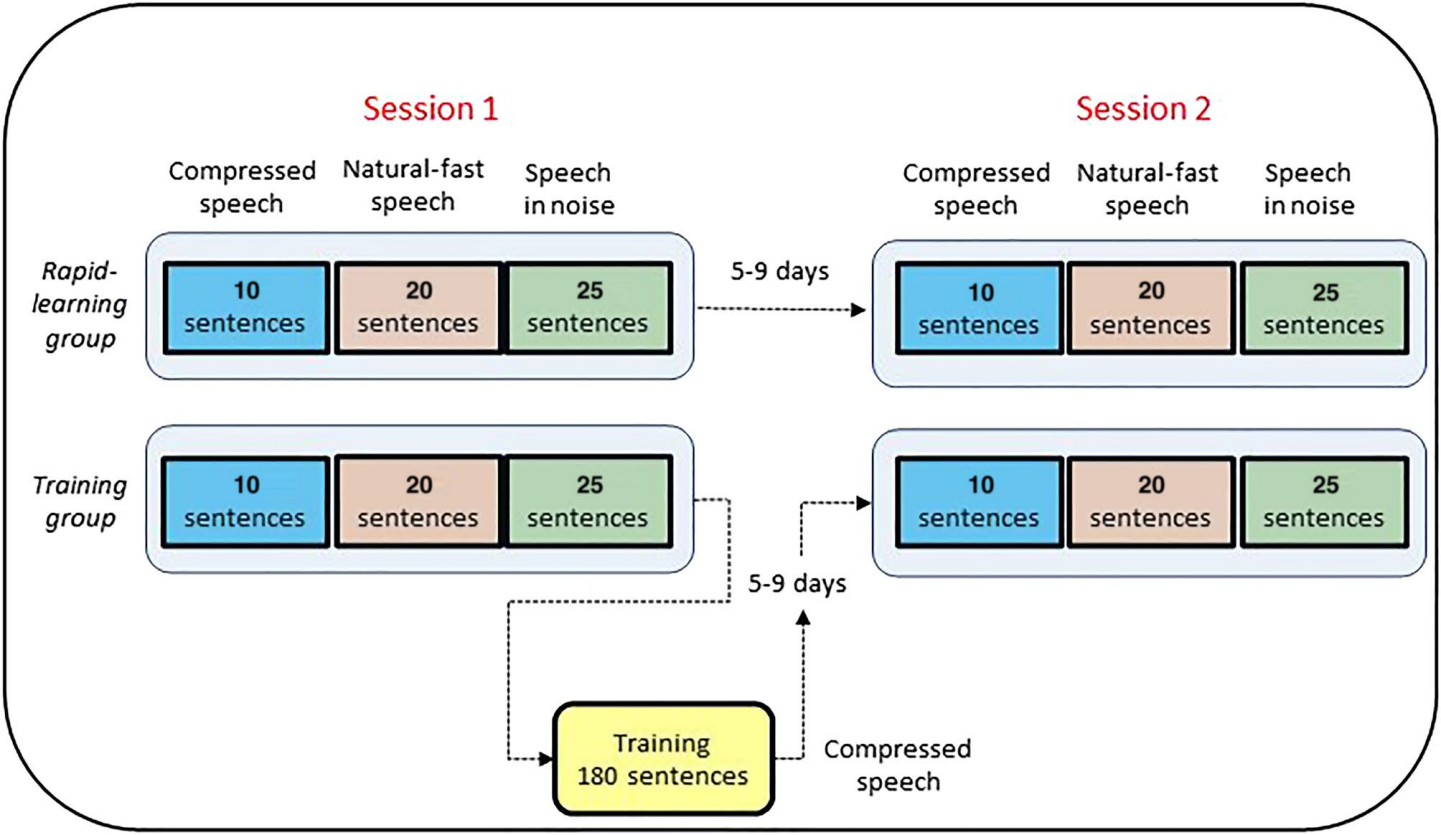
Overview of the design of Experiment 1. Each group completed two test sessions (Session 1, Session 2; the order of the three speech tests in each session was counterbalanced); The training group received additional training.

As shown in Fig. 1, the experiment comprised of two sessions, 5 to 9 days apart. On each session, all participants completed three speech recognition tests—time-compressed speech, natural-fast speech and speech-in-noise, in a counterbalanced order. The training group received additional training on time-compressed speech at the end of the first session. Participants completed the experiment in a quiet room on campus or in their homes. Stimuli were delivered to the two ears through headphones (Sennheiser HD-205 or HD-215) at a comfortable listening level, using custom software^44^. The time-compressed speech task was used to assess learning within and between sessions. Comparisons between the rapid-learning and the training groups were used to assess differences between rapid learning induced by the time-compressed speech tests and training-induced learning. The other two tasks were used to determine if perceptual learning of one type of speech is related to recognition of other types of challenging speech.

#### Stimuli and tasks

##### Stimuli

290 simple sentences in Hebrew (based on Prior and Bentin^51^), were used. Sentences were five to six words long and had a subject-verb-object grammatical structure. Half of the sentences were semantically plausible (e.g., “the talented poet wrote a poem”) and half the sentences were implausible (e.g., “the angry shopkeeper fired the rabbit”).

Stimuli for the speech-in-noise and time-compressed speech tests were recorded by Talker 1, a female native speaker of Hebrew with an average speech rate of 111 words/min (SD = 17). Stimuli for the natural-fast speech test were recorded by Talker 2, also a female, native speaker of Hebrew, at an average natural-fast rate of 214 words/minute (SD = 26) because prior testing^22^ suggested that natural-fast speech by Talker 1 was not fast enough to challenge university students who are native speakers of Hebrew. Sentences were recorded in a sound attenuating room at a sampling rate of 44.1 kHz, with a standard microphone, and edited in Audacity® software© 2.1.3 to remove remaining noise and equate root-mean-square (RMS) amplitude across sentences.

##### Speech recognition tests

Sentences were randomly divided across tests such that on each test half the sentences were plausible, and half were implausible. Different sentences were used on each test and session. Within a test, the order of the sentences was random but fixed across participants, with no sentence repetition. Sentence delivery was self-paced. Participants were asked to transcribe the sentences as accurately as they could, and the number of correctly transcribed words was counted for each sentence. Only perfectly transcribed words (ignoring homophonic spelling errors) were counted as correct. The proportion of correct words per sentence was used as an index of recognition accuracy. The order of the three tests was counterbalanced across participants.

##### Speech-in-noise tests

On each session participants had to transcribe 25 different sentences. Sentences produced by Talker 1 were mixed with 4-talker babble noise^44^ at a signal-to-noise ratio of –6 dB.

##### Natural-fast speech tests

On each session participants had to transcribe 20 different sentences produced by Talker 2.

##### Time-compressed speech tests

On each session participants transcribed 10 sentences produced by Talker 1. To afford isolation of the rapid learning effects, we used the minimal number of sentences thought to yield rapid learning in the majority of participants based on previous work^22^. Sentences were compressed to 30% of their natural duration using a WSOLA algorithm^52^.

##### Training

Three blocks of 60 sentences each produced by Talker 1 were delivered on Session 1. In the first block, participants had to transcribe sentences compressed to 30% of their natural duration, as described above. The additional two blocks were adaptive. For each sentence participants had to determine whether it was semantically plausible or not. This procedure was used to give participants extra training without overburdening them. In these adaptive blocks, initial compression was 50%. Subsequently a 2-down/1-up staircase procedure was used to adjust compression based on participants’ responses. The training phase took 30–45 min to complete.

The training phase itself was not the focus of this study, and detailed analyses of learning during this phase were published elsewhere^16,17,29,41^. However, to determine that training did elicit learning, we analyzed the data from the non-adaptive recognition block as follows: For each participant a learning curve was constructed based on average performance in 6 ‘mini-blocks’ of 10 sentences each, and the slopes of these curves were calculated. Slopes were positive in 75/82 participants, suggesting that most participants learned during this phase. The average slope (Mean = 0.024, SD = 0.020) was significantly larger than zero (t(81) = 11.20, p < 0.001) with a large effect size (Cohen’s d = 1.24).

#### Data analysis

Recognition accuracy data (proportions of correctly identified words) were analyzed in R^53^ with a series of generalized linear mixed models using the lme4 package^54^. Generalized models were use because they require fewer assumptions on the distributions of the data and are more suitable for proportion data^55^; mixed models were used because they are recommended for individual differences studies with language data^56,57^. Figures were created in Matlab (R2019b; https://www.mathworks.com/) and Microsoft Office 365 (https://www.office.com/).

##### Learning analysis

We used data from the time-compressed speech tests to assess rapid perceptual learning within and between sessions as well as training-induced learning (see “Results”). Learning between the two test sessions was our main index of learning because it manifests the retention of learning over time. To this end, for each participant the proportion of words correctly transcribed across all sentences within a session was averaged and the difference between the averages of the two sessions served as a learning index. For the rapid-learning group, this is an index of the rapid learning induced by completing the tests. For the training group, the value is a mixture of the rapid learning that occurred during the tests and the additional contribution of training-induced learning. Group effects in the statistical models described in the Results were used to statistically separate rapid and training-induced learning. Within-session learning across sentences was also modeled to further assess rapid learning and how it may interact with training-induced learning.

### Results

#### Rapid learning of time-compressed speech is perceptual

**Figure 2:**
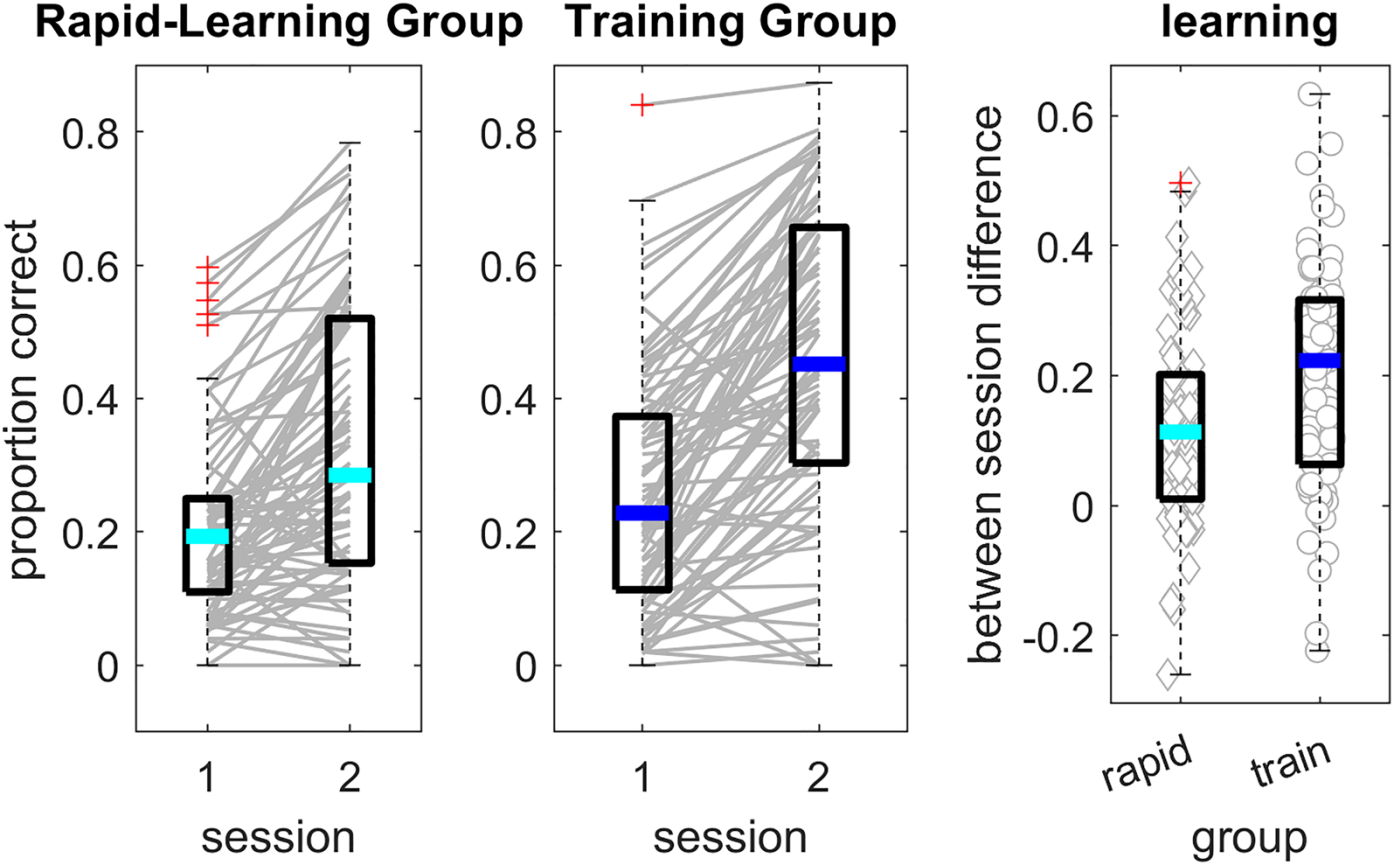
Time-compressed speech recognition and learning in the rapid-learning and training groups. The left and center panels show performance averaged within each participant in each session. Lines show data for individual participants. The rightmost panel show learning, expressed as the difference in recognition accuracy between the two sessions in the rapid-learning group (left) and in the training group (right). Background symbols denote individual data. The thick line within each boxplot shows group median; box edges mark the interquartile range; whiskers are 1.5 times the interquartile range; + signs are values outside the 1.5 × interquartile range.

Time-compressed speech recognition in the two groups and sessions is shown in Fig. 2. In the rapid-learning group, mean recognition accuracy was 0.20 (SD = 0.14) in session 1, and 0.33 (SD = 0.21) in session 2. In the training group, mean accuracy was 0.26 (SD = 0.18) in session 1 and 0.47 (SD = 0.22) in session 2. Our first goal was to determine whether learning of time-compressed speech occurred between the two sessions and whether it differed between the two groups. Learning, defined as the amount of improvement on time-compressed speech recognition accuracy between the two sessions, is also shown in Fig. 2. It suggests that recognition accuracy of the majority of participants in both groups improved between the two sessions.

To determine whether this learning was significant, and whether it was modulated by additional practice, mixed modelling was conducted. Random effects included random intercept for participants, as well as a sentence by participant random slope to account for the possibility that learning rates (changes in accuracy over sentences) vary across participants. Fixed effects included group (*rapid-learning*, dummy coded as 0 and *training*, coded as 1), sentence number (coded 1 to 10) and session (session 1 coded 0 and session 2 coded as 1). A binomial regression with logistic link function was used (as recommended for proportion data^55^). Three models were constructed. A model that included the random effects only (AIC = 11,485), a model with additional main effects for each of the three fixed factors (AIC = 10,670), and a “full” model that included all possible interaction terms between the fixed factors (AIC = 10,558). Model comparisons (using the anova() function in R) suggested that the model with main effects fits the data significantly better than the model with random effects only (χ^2^_(3)_ = 821, p ≤ 0.001) and the full model fits the data better than the model with only main effects (χ^2^_(4)_ = 120, p ≤ 0.001).

**Figure 3:**
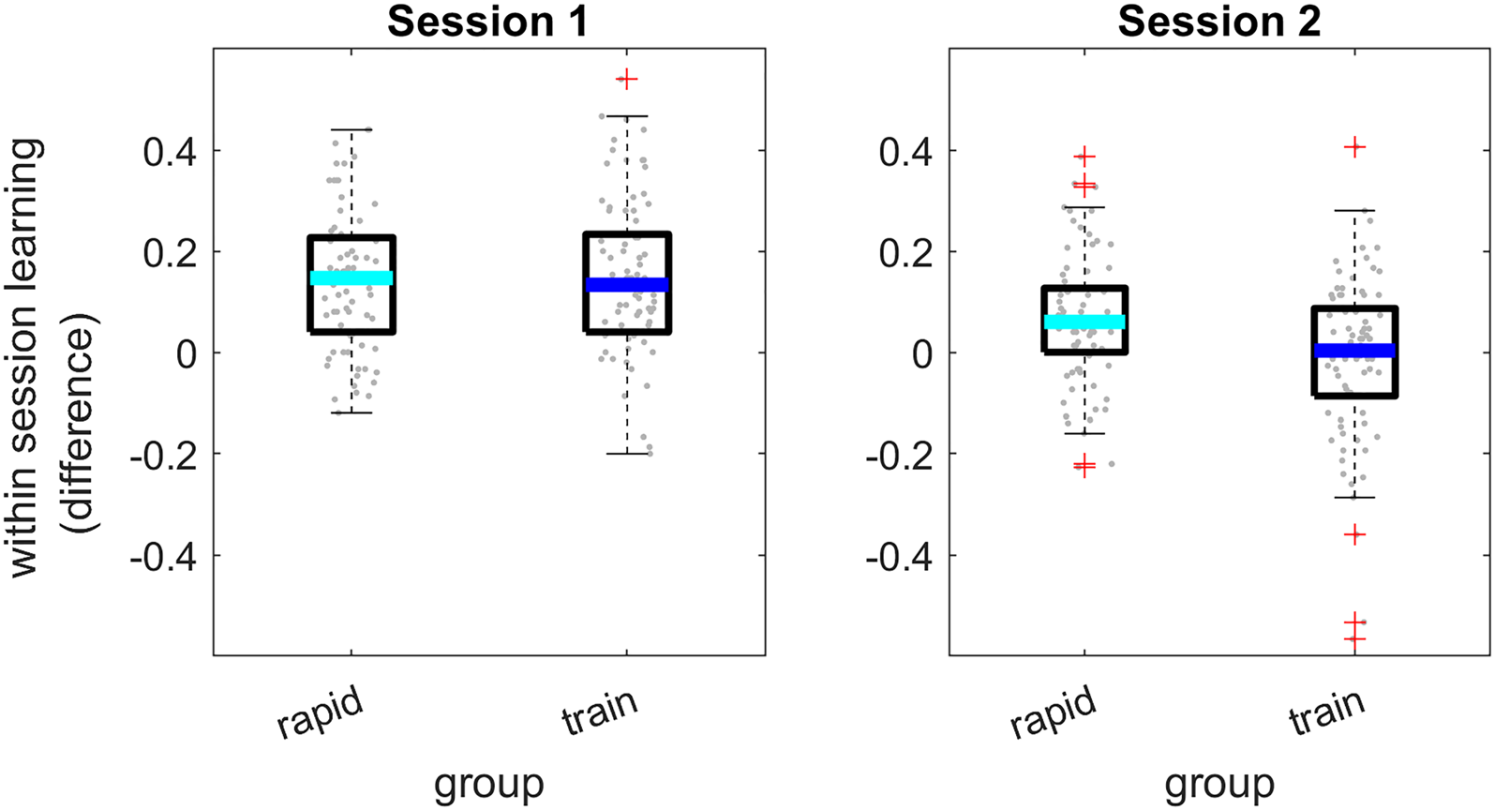
Rapid (within-session) learning as a function of group. Rapid learning, defined as the difference in performance accuracy between the first 5 and last 5 time-compressed sentences on each session. rapid = rapid-learning group; train = training group. Background symbols denote individual data. The thick line within each boxplot shows group median; box edges mark the interquartile range; whiskers are 1.5 times the interquartile range; + signs are values outside of the 1.5 × interquartile range.

The effects in the full model (see Table 1) were used to determine whether learning occurred, whether it was maintained over time and whether it differed between the two groups. As expected, a significant main effect of sentence was present, confirming that rapid learning of time-compressed speech occurred within session. Overall performance was more accurate in the second session (main effect of session). A test of simple main effects confirmed that accuracy increased in both groups (rapid-learning: estimate = 0.82, Z = 14.75, p < 0.001; training: estimate = 1.15, Z = 22.25, p < 0.001). Between-session improvements were larger in the training group (significant group by session interaction), suggesting that training resulted in greater learning than brief exposure. On the other hand, the magnitude of learning within a session was smaller in the second session (significant sentence by session interaction). Although Fig. 3 suggests that the magnitude of decline in rapid learning between sessions could have been larger in the training group, the group by session by sentence interaction was not significant.

**Table 1:** Fixed effects and interactions from the full learning model.

Effect	β	SE	Z	p
Group	0.43	0.24	1.8	0.072
Session	1.34	0.13	10.69	≤ 0.001
Sentence	0.19	0.02	10.89	≤ 0.001
Group × session	0.61	0.17	3.61	≤ 0.001
Group × sentence	− 0.01	0.02	− 0.53	0.594
Sentence × session	− 0.10	0.02	− 5.00	≤ 0.001
Group × session × sentence	− 0.05	0.03	− 1.95	0.051

To help interpret the effects from the statistical model, within-session learning is presented in Fig. 3. Each listener transcribed 10 (different) time-compressed sentences on each session, and learning was defined as the difference in transcription accuracy between the final and first 5 sentences in a session. Figure 3 suggests that on the first session this learning was larger than zero (t(161) = 12.87, p < 0.001, Cohen’s d = 1.01) with no significant difference between the two groups (Mean = 0.14 and 0.15; SD = 0.14 and 0.15 in the rapid-learning and training groups respectively t(160) = − 0.43, p = 0.623). As suggested from the sentence by session interaction in the full model, on the second session, learning was still significant (t(161) = 2.42, p = 0.008) but of smaller magnitude (Cohen’s d = 0.19; Mean = 0.07 and − 0.01, SD = 0.13 and 0.16, in the two groups respectively). While the group by sentence by session interaction was not significant in the full model, on the second session learning was significant in the rapid-learning (t(79) = 4.7, p < 0.001; Cohen’s d = 0.53) but not in the training (t(81) = − 0.51, p = 0.61, Cohen’s d = − 0.06) group. Furthermore, whereas learning during the second session was observed in 56/79 participants in the rapid-learning group (with a median of 0.06 and interquartile range from 0 to 0.13), only 41/80 participants in the training group continued to improve during session 2 (Median = 0.003, IQR = − 0.087 to 0.087; χ^2^ = 6.44, p = 0.011).

Taken together, these data suggest that rapid learning occurred during the first test session, and to a lesser extent during the second session in participants that did not receive additional training (the rapid-learning group); additional training resulted in additional learning between sessions, and reduced rapid learning in the second session. Furthermore, rapid learning was maintained between sessions, conforming to the definition of perceptual learning.

#### Rapid learning and individual differences in speech recognition

One of the goals of this experiment was to determine whether perceptual learning of time-compressed speech was associated with speech perception in independent tasks (natural- fast speech and speech-in-noise), and if so, whether rapid- and training-induced learning differed in this respect.

**Figure 4:**
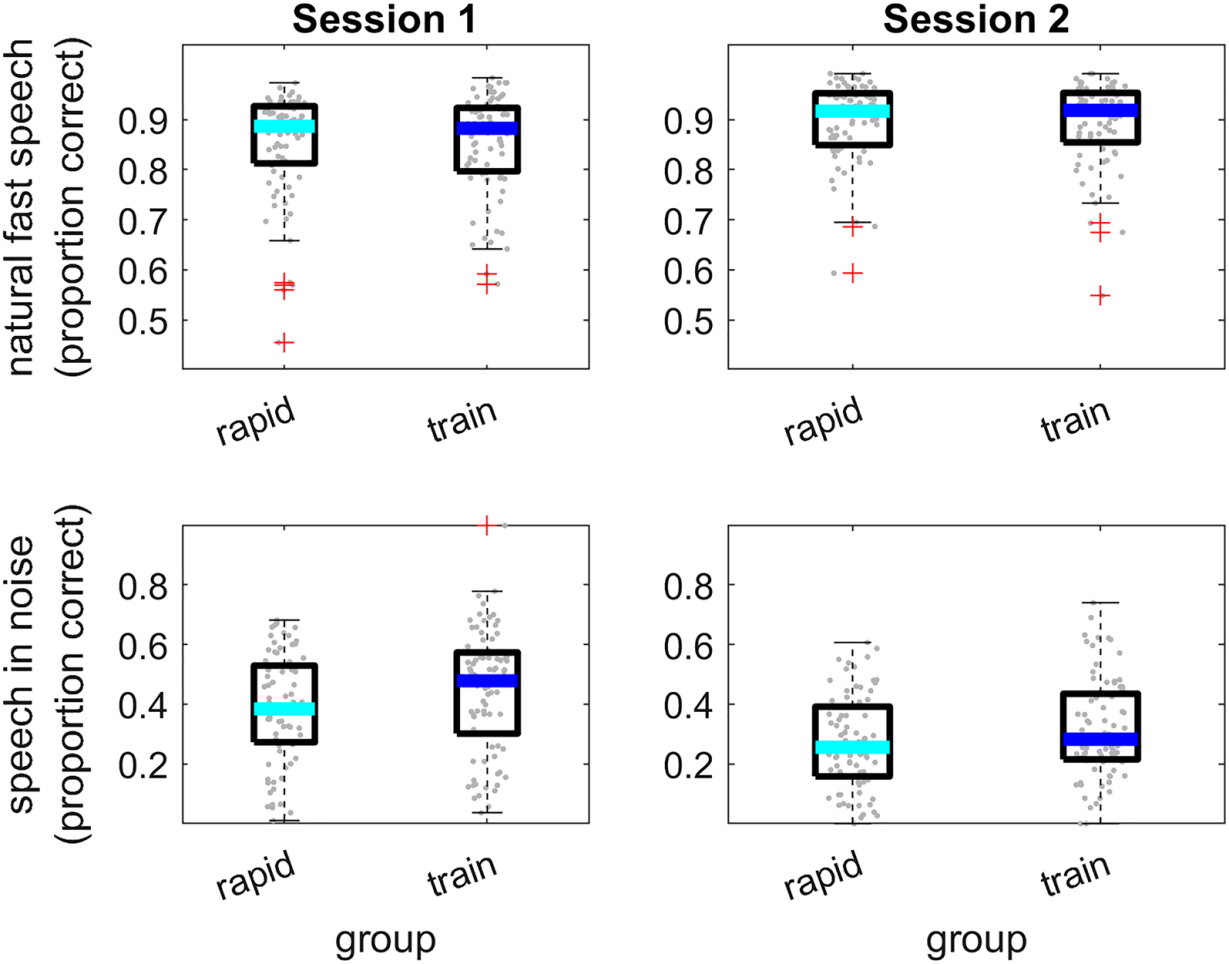
Perception of natural-fast speech and speech-in-noise as a function of group and session. rapid = rapid-learning group; train = training group. Background symbols denote mean individual data across sentences. The thick line within each boxplot shows group median; box edges mark the interquartile range; whiskers are 1.5 times the interquartile range; + signs are values outside of the 1.5 × interquartile range.

Speech perception in the two groups and sessions is shown in Fig. 4 (Natural-fast speech: Session 1: Mean = 0.86, SD = 0.10 and Mean = 0.85, SD = 0.09; Session 2: Mean = 0.89, SD = 0.09 and Mean = 0.89, SD = 0.11 in the rapid-learning and training groups, respectively; Speech-in-noise: Session 1: Mean = 0.41, SD = 0.15 and Mean = 0.46, SD = 0.20; Session 2: Mean = 0.30, SD = 0.15 and Mean = 0.35, SD = 0.16 in the rapid-learning and training groups, respectively). Similar performance in the two groups in Session 2 would suggest that the training provided to the training group during Session 1 had no significant contribution to performance in those tasks, and thus that associations between between-session learning on time-compressed speech and Session 2 performance on the other speech tasks reflect rapid perceptual learning. Therefore, speech perception data in each task was modelled as a function of group, session and group by session interaction as fixed effects and random intercepts for participants and individual sentences. Model comparisons suggested that the model with all fixed effects (AIC = 13,832) was a better fit to the natural-fast speech data than the model with random effects only (AIC = 13,939; χ^2^_(3)_ = 112, p ≤ 0.001). The fixed effects (see Table 2) suggested that natural-fast speech recognition was more accurate in Session 2, but as both the group effect and the session by group interaction were insignificant, this is not due to generalization of training-induced learning of time-compressed speech in the training group. Similarly, for speech-in-noise, model comparisons suggested that the model with fixed effects (AIC = 25,334) was a better fit for the data than the model with random effects only (AIC = 25,959; χ^2^_(3)_ = 631, p ≤ 0.001). Although speech-in-noise recognition was poorer in Session 2 than in Session 1, there was no indication that this is due to training (see Table 2). Therefore, Session 2 speech perception data were used in the following analyses to assess the associations between perception and learning of time-compressed speech.

**Table 2:** Natural-fast speech and speech-in-noise perception as a function of group and session.

Effect	Natural-fast speech				Speech-in-noise			
	β (log-odds)	SE	Z	p	β (log-odds)	SE	Z	p
Group	− 0.03	0.13	− 0.24	0.808	0.23	0.14	1.66	0.097
Session	0.37	0.05	7.59	≤ 0.001	− 0.59	0.03	− 18.51	≤ 0.001
Group × session	− 0.01	0.07	− 0.21	0.830	0.07	0.04	1.58	0.11

**Figure 5:**
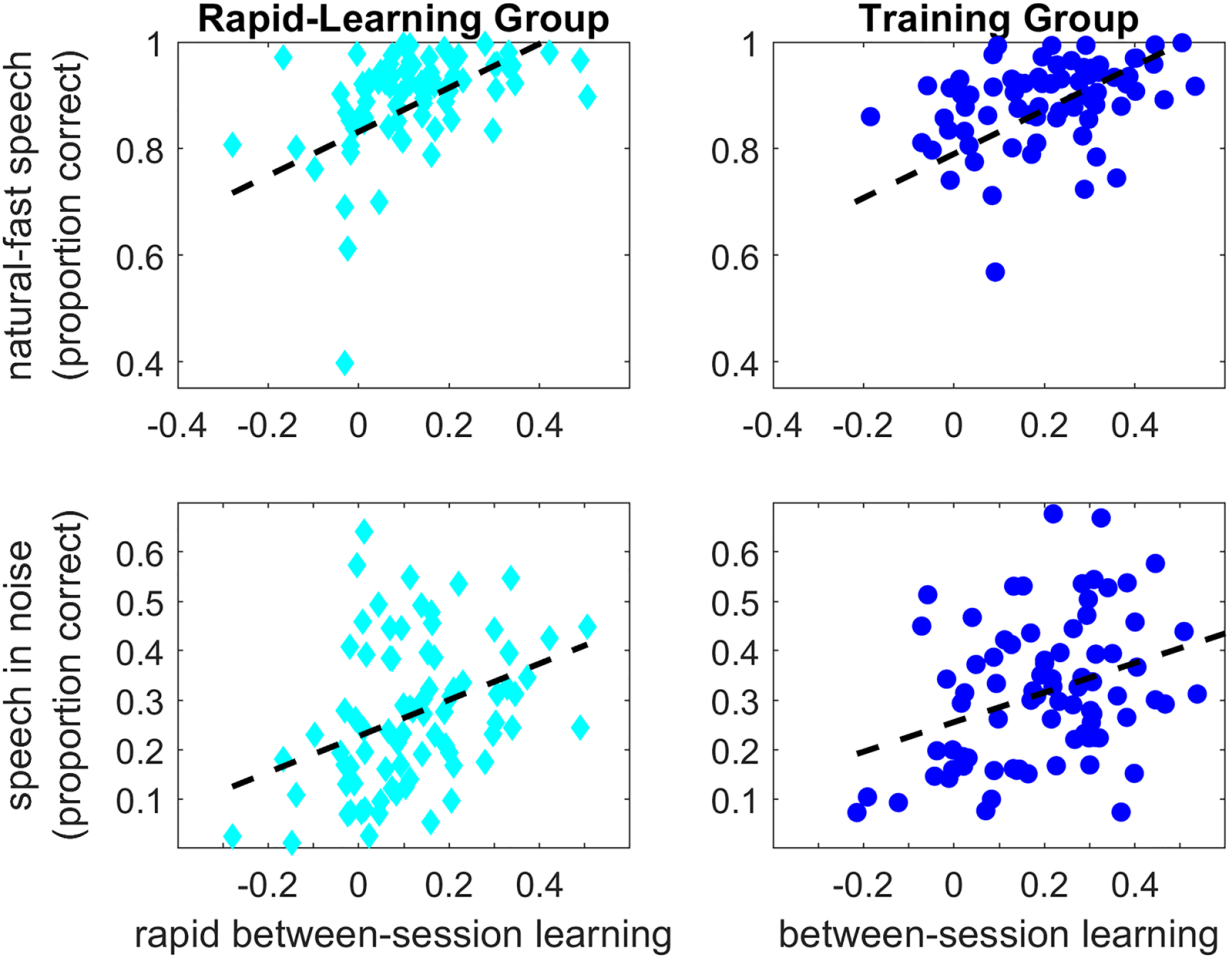
Speech recognition versus perceptual learning. Proportions correct in session 2 are plotted against between-session learning of time-compressed speech expressed as the difference in recognition accuracy between the two sessions for each participant. Dashed lines show linear fits. For visualization only, learning scores were adjusted to partial out the contribution of baseline recognition of time-compressed speech (as in Manheim et al., 2018). Therefore, values on the x axes are not the same as the simple difference scores shown in Fig. 2.

Speech recognition is plotted in Fig. 5 as a function of perceptual learning. To determine how perceptual learning contributed to speech recognition in the two tasks, data was again modelled with mixed-effects binomial regression with a logistic link function. For each speech task, the following models were constructed: (1) a “random” model with random intercepts for participant and sentence; (2) a “main effects” model which included three additional main effects: group (rapid-learning coded as 0 and training coded as 1), perceptual learning (the difference between Session 2 and Session 1 as plotted in Fig. 2) and baseline recognition of time-compressed speech (mean of the first 5 sentences from session 1); the two continuous predictors were scaled , and (3) an “interaction” model in which the group by learning interaction was also included. Model comparisons were used to determine whether the “main effects” model fits the speech data better than the model with random effects only. Then the “main effect” and “interaction” models were compared to determine if the contribution of perceptual learning to speech perception differed between the rapid-learning and training groups.

For natural-fast speech, the “main effects” model (AIC = 5991) significantly improved data fit over the “random” model (AIC = 6030, χ^2^_(3)_ = 45, p ≤ 0.001). Adding the group by learning interaction in the “interaction” model had no significant effect (AIC = 5993, χ^2^_(1)_ = 0.69, p = 0.406, see Table 3 for the parameters of the best fitting model). For speech-in-noise, the “main effects” model (AIC = 11,538) fitted the data significantly better than the random model (AIC = 11,586, χ^2^_(3)_ = 54, p ≤ 0.001). Addition of the group by learning interaction had no significant impact on the fit (AIC = 11,539, χ^2^_(3)_ = 0.77, p = 0.381, see Table 3 for the parameters of the best fitting model).

**Table 3:** Prediction models for speech recognition as a function of perceptual learning.

Effect	Natural-fast speech				Speech-in-noise			
	β log-odds	SE	Z	p	β log-odds	SE	Z	p
Group	− 0.41	0.14	− 2.82	0.005	0.04	0.13	0.34	0.737
Learning	0.42	0.07	6.02	≤ 0.001	0.31	0.07	4.63	≤ 0.001
Baseline	0.31	0.07	4.41	≤ 0.001	0.40	0.06	6.28	≤ 0.001

Together, it seems that perceptual learning of time-compressed contributes to the recognition of natural-fast speech or speech-in-noise. Additional training did not modify these associations significantly (insignificant group by learning interactions). While similar associations were reported before for within-session learning^22^, the current findings suggest that the association reflects perceptual learning that is retained over time rather than transient effects. Furthermore, the current findings suggest that the contribution of perceptual learning is not attributable to generalization across speech tasks.

## Experiment 2

The outcomes of Experiment 1 suggest that rapid perceptual learning of time-compressed speech is associated with individual differences in other speech tasks. They also suggest that additional training yields additional learning on the trained task. On the other hands, the characteristics of rapid learning, and particularly its stimulus specificity are not well understood. Both talker variability and stimulus repetition were previously suggested to influence the specificity of perceptual learning for speech^58–60^. Although we found no effect for either of these factors in past training studies with time-compressed speech^17,29^, they could still influence more rapid learning on this task. Experiment 2 therefore explored the effects of repetition (5 repetitions of each of 4 sentences and 20 repetitions of a single sentence) and talker variability (1 vs. 5 talkers) on rapid learning of time-compressed speech and its talker specificity.

### Methods

#### Participants

254 native Hebrew speakers (ages 18–35 years, Mean = 27, SD = 4; 153 females, 102 males) participated in this study. All other details are as in Experiment 1. Participants were randomly divided to five groups and tested as described below; No other tests were conducted.

#### Overview of the experiment and rapid-learning groups

**Figure 6:**
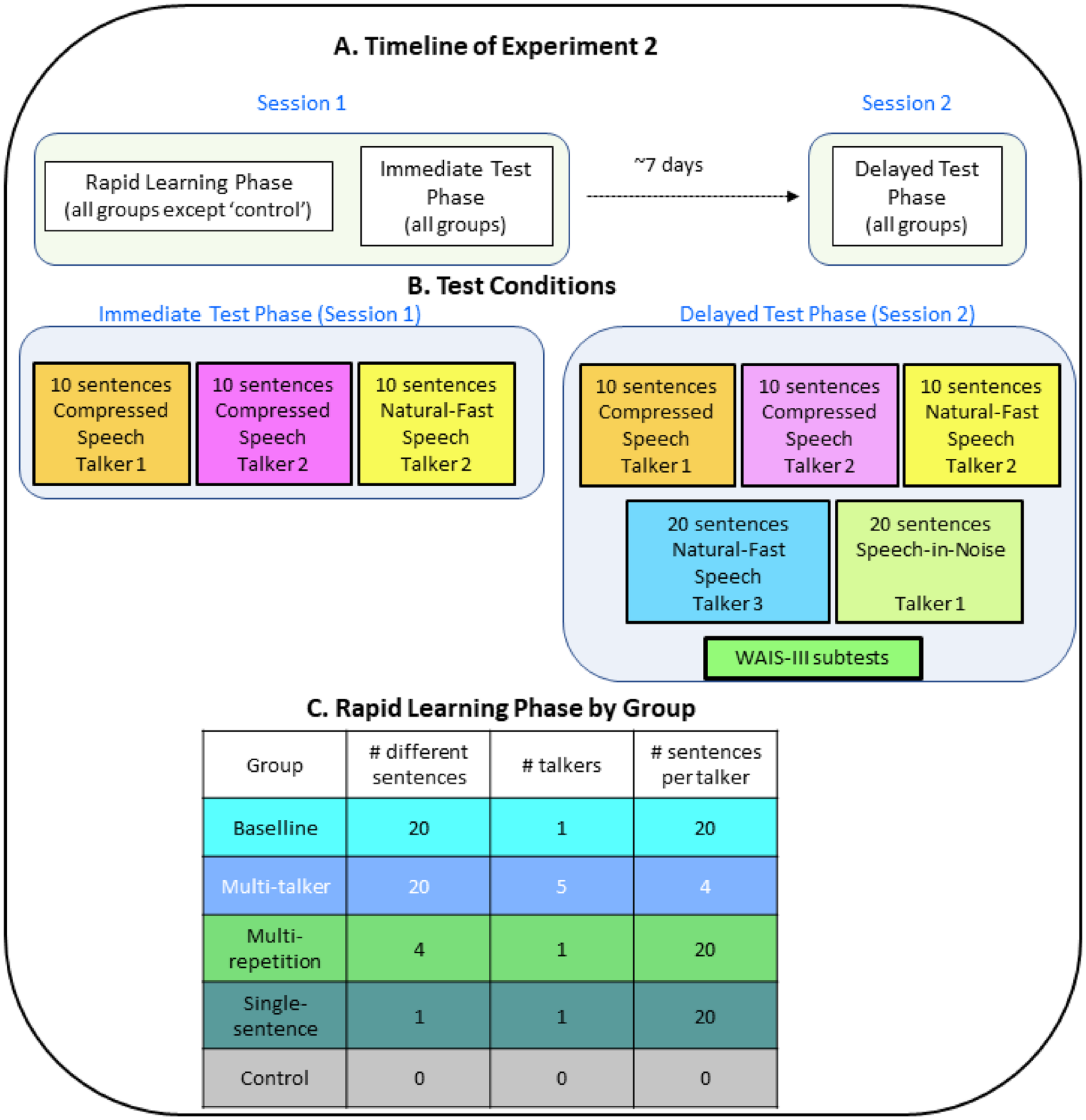
Experiment 2 design. A. Timeline. B. Test conditions. C. Composition of the stimulus set during the rapid-learning phase.

Participants were assigned randomly to one of five groups, a ‘no exposure’ control group and four rapid-learning groups and participated in two sessions (see Fig. 6A). During the first session, participants in the rapid-learning groups completed a rapid-learning phase. Subsequently, all groups completed the immediate test phase. On the second session, approximately one week later, all participants completed the delayed test phase (Fig. 6B). During the rapid-learning phase each of the rapid-learning groups listened to and transcribed 20 time-compressed sentences that differed in the number of talkers presenting the sentences and the number of times each individual sentence was presented (see Fig. 6C): ‘baseline’ (20 different sentences presented by a single talker), ‘multi-talker’ (the same 20 sentences presented by 5 different talkers such that each talker delivered 4 different sentences), ‘multi-repetition’ (four sentences presented by a single talker, each repeated five times), and ‘single sentence’ (one sentence presented 20 times by a single talker).

On the first session, participants in the rapid-learning groups transcribed 20 time-compressed sentences (Fig. 6). After the exposure, all five groups were tested on the time-compressed and natural-fast speech tests described below in a fixed order. On the second session (~ 7 days after session 1) they were again tested on the same tests (with different sentences), again in fixed order. Then they completed another natural-fast speech test, a speech-in-noise test and the matrices, digit-span and similarities subtests from WAIS-III^61^ in counterbalanced order.

#### Stimuli, rapid-learning and test conditions

120 simple sentences in Hebrew were used in this study (see Experiment 1 for further details). Sentences for the exposure condition were recorded with five native speakers of Hebrew (all female), including Talkers 1 and 2 from Experiment1. Natural-fast speech was recorded by Talker 2 and an additional Talker 3, also a female native speaker of Hebrew with an average speech rate of 220 words/minute (SD = 21).

##### Rapid-learning conditions

In all groups listeners had to transcribe 20 sentences compressed to 30% of their original duration as in Experiment 1. No feedback was provided (see Fig. 6).

##### Baseline

20 different sentences were presented by Talker 1. This condition is similar to those used in past studies to document rapid learning of time-compressed speech^22,28,32^.

##### Multi-talker

The same sentences as in the baseline condition were presented by five different talkers (as in^29^, except all talkers were female and native speakers of Hebrew), including Talker 1 and Talker 2. The remaining talkers were not presented in any other phase of this experiment.

##### Multi-repetition

Four sentences were selected randomly from the baseline condition and presented five times each by Talker 1 in pseudo-random order such that a single sentence could not repeat on two successive trials.

##### Single sentence

To further probe the effects of stimulus repetition, a single sentence was randomly selected from the baseline condition and presented 20 times by Talker 1. The same sentence was played to all participants.

##### Test conditions (see Fig. 6)

On each test session participants had to transcribe 10 time-compressed sentences presented by Talker 1, 10 time-compressed sentences presented by Talker 2 and 10 natural-fast sentences presented by Talker 2, in this fixed order. In Session 2, after completing these tests, two other speech tests were carried out. In one test, 20 natural-fast sentences recorded by Talker 3 were presented as another test of potential group differences with a new talker. In the other, 20 sentences recorded by Talker 1 and embedded in background noise (as in Experiment 1) were presented. In addition, we administered three subtests from WAIS-III (see Table 4). The WAIS-III subtests and the additional speech tasks were presented in counterbalanced order. These were included to rule out group differences in cognition incase significant differences emerged as a function of rapid learning.

**Table 4:** Group characteristics—mean (SD).

	No exposure	Baseline	Multi-talker	Multi-Repetition	Single sentence
N	53	51	50	50	50
F:M	31:22	28:23	35:15	31:19	27:23
Age	27 (4)	27 (4)	27 (4)	26 (4)	26 (4)
Education	14 (2)	14 (2)	14 (2)	14 (2)	14 (2)
Days between sessions	7 (1)	7 (1)	7 (1)	7 (1)	7 (1)
Digit span	12.6 (3)	12.2 (3)	11.5 (3)	12.1 (3)	11.3 (3)
Similarities	10.9 (2)	11.2 (3)	11.3 (2)	11.0 (2)	11.2 (2)
Matrices	14.1 (2)	14.2 (2)	13.9 (2)	13.7 (3)	13.8 (2)

Education is given in years. For Digit span, Similarities and Matrices scaled scores are reported.

##### Sentence transcription

Across exposure and testing, presentation was self-paced. Listeners heard each sentence, transcribed it and continued to the next sentence by pressing a “continue” button on screen using custom software^44^. Each sentence was played once, and no feedback was provided.

#### Data analysis

For each test sentence, the proportion of correctly transcribed words was calculated and submitted for further analysis. As in Experiment 1, data were analyzed using mixed effects generalized linear modelling (using lme4 in R) with random intercepts for sentence and participant. Proportions of correct responses on each test were the dependent variables. Rapid-learning group (coded 0, 1, 2, 3, 4 for the control, baseline, multi-talker, multi-repetition and single sentence conditions, respectively) and test session (Session 1, Session 2) were fixed effects for the time-compressed and Talker 2 natural-fast speech tests. Exposure condition was the only fixed factor for Talker 3 natural-fast speech and for the speech-in-noise test which were conducted in Session 2 only. For each dependent variable (Talker 1, Talker 2 etc.), model comparisons were used to determine whether the inclusion of each of the fixed effects improved the fit of the model significantly.

### Results

#### Test performance as a function of rapid learning

**Figure 7:**
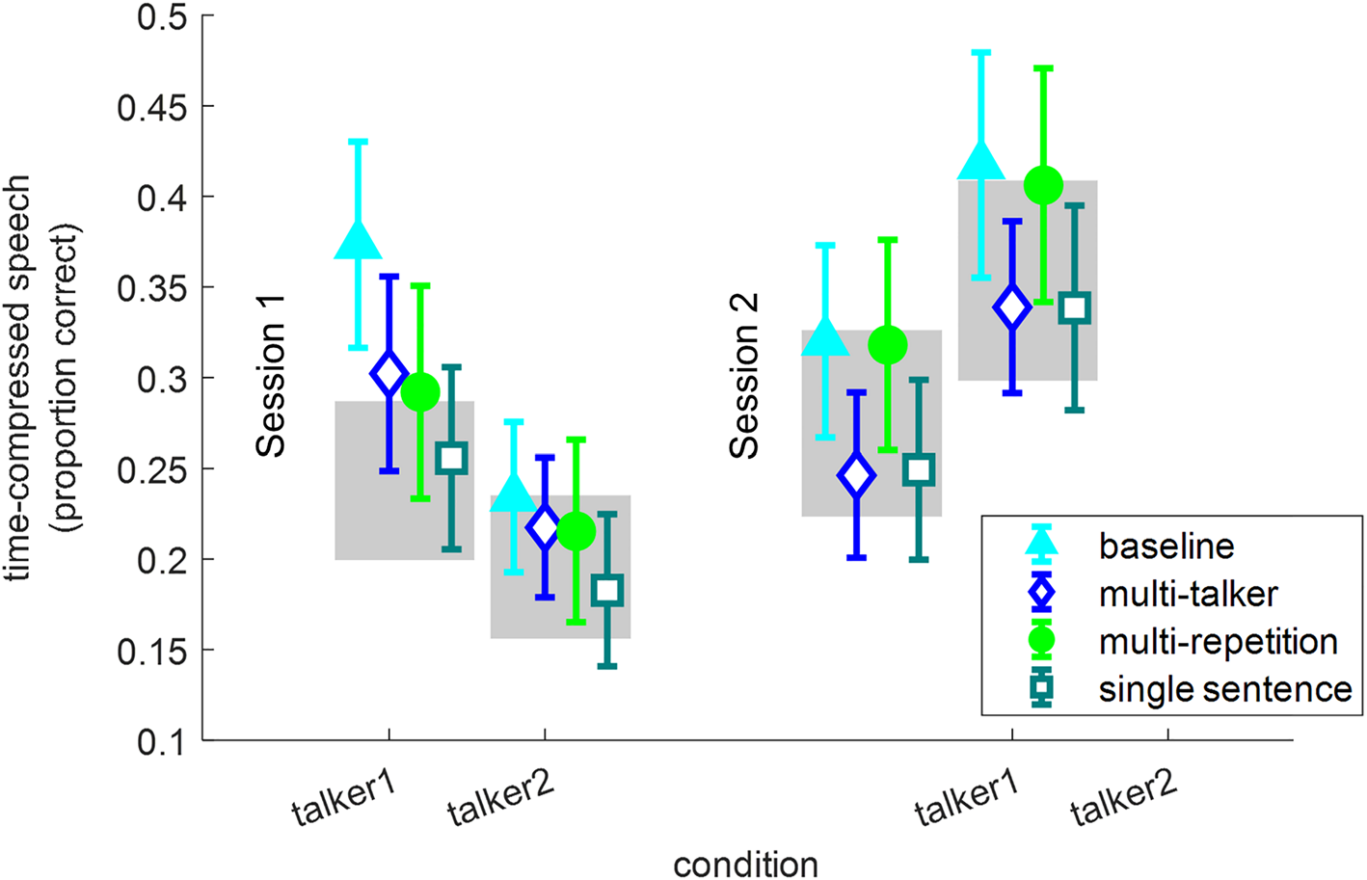
Time-compressed speech recognition by rapid-learning condition in the immediate (Session 1) and delayed (Session 2) tests. For each rapid-learning group the mean (across all sentences per condition) and 95% confidence interval are shown. The grey rectangles mark the 95% confidence interval of the control group who participated in testing only.

Time-compressed speech recognition accuracy is shown in Fig. 7 and in Table 5 for each group and test.

**Table 5:** Time-compressed speech and natural-fast speech by exposure condition and session.

	Time-compressed talker 1		Time-compressed talker 2		Natural-fast talker 2	
	Mean (SD)	Median (IQR)	Mean (SD)	Median (IQR)	Mean (SD)	Median (IQR)
**Control group**						
Session 1	0.24 (0.16)	0.23 (0.12–0.35)	0.19 (0.14)	0.16 (0.11–0.27)	0.58 (0.14)	0.60 (0.49–0.66)
Session 2	0.28 (0.19)	0.24 (0.14–0.41)	0.35 (0.20)	0.34 (0.21–0.49)	0.68 (0.13)	0.70 (0.62–0.75)
**Baseline group**						
Session 1	0.37 (0.20)	0.41 (0.25–0.50)	0.23 (0.15)	0.22 (0.10—0.37)	0.56 (0.16)	0.61 (0.47–0.68)
Session 2	0.32 (0.19)	0.31 (0.19–0.45)	0.42 (0.22)	0.43 (0.28–0.54)	0.67 (0.16)	0.72 (0.62–0.78)
**Multi-talker group**						
Session 1	0.30 (0.19)	0.31 (0.13–0.42)	0.22 (0.14)	0.20 (0.09–0.32)	0.54 (0.15)	0.57 (0.41–0.65)
Session 2	0.25 (0.16)	0.21 (0.15–0.31)	0.34 (0.17)	0.32 (0.20–0.45)	0.65 (0.14)	0.70 (0.56–0.76)
**Multi-repetition group**						
Session 1	0.29 (0.21)	0.27 (0.11–0.42)	0.22 (0.18)	0.16 (0.08–0.34)	0.50 (0.16)	0.55 (0.40–0.62)
Session 2	0.32 (0.20)	0.29 (0.17–0.45)	0.41 (0.23)	0.37 (0.24–0.55)	0.68 (0.18)	0.71 (0.62–0.81)
**Single sentence group**						
Session 1	0.26 (0.18)	0.24 (0.13–0.37)	0.18 (0.15)	0.15 (0.06–0.27)	0.52 (0.16)	0.55 (0.45–0.60)
Session 2	0.25 (0.17)	0.22 (0.11–0.33)	0.34 (0.20)	0.31 (0.19–0.45)	0.67 (0.13)	0.68 (0.62–0.75)

For Talker 1, each successive model fit the data better than the previous one. The model with exposure condition (AIC = 15,397) fit the data better than the model with random effects only (AIC = 15,399, χ^2^_(4)_ = 9.54, p = 0.049). Adding session reduced AIC to 15,392 (χ^2^_(1)_ = 7.31, p = 0.007). The model with interactions fit the data best (AIC = 15,344, χ^2^_(4)_ = 55.78, p ≤ 0.001). However, this model was hard to interpret because as shown in Fig. 7, performance in the control group (marked with grey patches on the figure) improved between the immediate and delayed tests, whereas changes in the rapid-learning groups were variable: performance decayed in the ‘baseline’, ‘multi-talker’ and ‘single-sentence’ groups and somewhat increased in the ‘multi-repetition’ group. An inspection of the model parameters (Table 6) suggests that the condition by session interaction stems from a decrease in group difference between the baseline and the control groups, the multi-talker and the control groups and the single-sentence and the control groups, from Session 1 to Session 2. In other words, the experience that the control group received by participating in the test sessions resulted in improvements. Consequently, when the other groups were compared with the control group, the effects of learning were diminished.

**Table 6:** Prediction models for time-compressed speech recognition as a function of group, talker and session (including group × session interactions).

Parameter	Talker 1		Talker 2	
	Log-odds (SE)	Z (p)	Log-odds (SE)	Z (p)
Intercept (control)	− 1.44 (0.23)	− 6.23 (< 0.001)	− 1.86 (0.29)	− 6.49 (< 0.001)
Baseline	0.75 (0.22)	3.50 (< 0.001)	0.26 (0.22)	1.21 (0.224)
Multi-talker	0.41 (0.22)	1.90 (0.058)	0.21 (0.22)	0.97 (0.334)
Multi-repetition	0.28 (0.22)	1.29 (0.197)	0.13 (0.22)	0.57 (0.566)
Single sentence	0.04 (0.22)	0.18 (0.853)	− 0.12 (0.22)	− 0.56 (0.577)
Session 2	0.18 (0.07)	2.73 (0.006)	1.03 (0.07)	14.84 (< 0.001)
Baseline × Session 2	− 0.48 (0.09)	− 5.20 (< 0.001)	0.09 (0.10)	0.90 (0.371)
Multi-talker × Session 2	− 0.54 (0.09)	− 5.73 (< 0.001)	− 0.26 (0.10)	− 2.64 (0.008)
Multi-repetition × Session 2	− 0.04 (0.09)	− 0.38 (0.701)	0.18 (0.10)	1.81 (0.070)
Single sentence × Session 2	− 0.23 (0.10)	− 2.41 (0.016)	0.05 (0.10)	0.05 (0.613)

For Talker 2, including group in the model did not significantly improve the fit of the model to the data (AIC = 16,234 in the model with random effects only, AIC = 16,237 in the model with rapid-learning group as a fixed effect, χ^2^_(4)_ = 4.94, p = 0.293), but the addition of session (AIC = 15,103, χ^2^_(1)_ = 1135, p < 0.001) and the rapid-learning group by session interaction (AIC = 15,089, χ^2^_(4)_ = 22.32, p < 0.001) did. However, as all groups improved from Session 1 to Session 2, it seems that learning during the tests was sufficient for learning the time-compressed speech produced by Talker 2 regardless of previous exposure (see Table 6).

**Figure 8:**
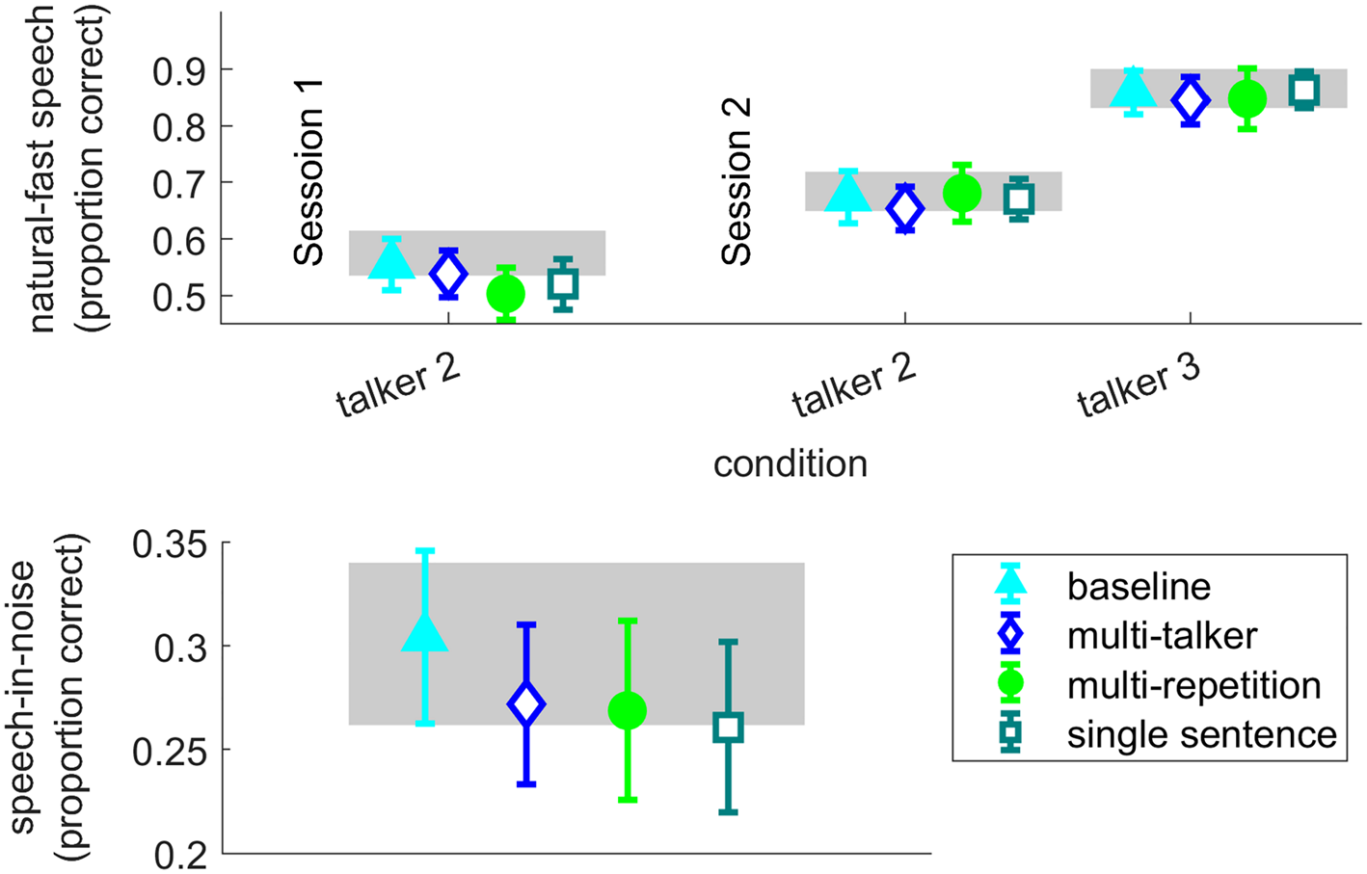
Natural-fast speech (top, left to right—Talker 2 in Session 1, Talker 2 in Session 2 and Talker 3 in Session 2) and speech-in-Noise (bottom) recognition (tested in Session 2 only). For each rapid-learning group mean (across all sentences per condition) and 95% confidence interval are shown. The gray rectangles mark the 95% confidence interval of the control group who participated in testing only.

Finally, there were no group differences in the recognition of either natural-fast speech (Talker 3) or speech-in-noise in the delayed test in Session 2 (Fig. 8). For natural- fast speech, adding rapid-learning group had no significant effect on the fit compared to a model with random effects for item and participant only (AIC = 10,681, AIC = 10,688 in the model with random effects only and the model with group respectively, χ^2^_(4)_ = 0.67, p = 0.955). The same is true for speech-in-noise (AIC = 13,941 in the random model, AIC = 13,945 in the model that included rapid-learning condition, χ^2^_(4)_ = 3.64, p = 0.456). Given the group data shown in Table 5 and Fig. 8 (top panel), we decided not to model that natural-fast speech data from talker 2 for group differences.

## Discussion

Active listening to 10 time-compressed sentences was sufficient for robust and long-lasting perceptual learning (Experiment 1), consistent with previous findings on within-session learning^22,28^. This rapid learning was specific to the acoustic characteristics of the speech used to elicit learning (Experiment 2). Although additional practice resulted in more learning, the associations between perceptual learning and speech recognition were driven by rapid learning (Experiment 1). In the context of previous work (e.g.,^5,29,37,41^) these data tentatively suggest that additional practice does not change the nature of the resulting perceptual learning. If this is the case, rapid learning is key in understanding the function of perceptual learning in speech recognition, as we discuss in the following sections.

### Long-lasting and specific: the outcomes of rapid learning are consistent with the characteristics of perceptual learning

In the current study, the amount of practice received (rapid learning only vs. three blocks of training in Experiment 1) had quantitative but not qualitative effects on perceptual learning. Consistent with previous studies^29,32^, rapid learning was relatively long lasting (Experiment 1), but also quite specific to the acoustics of the stimuli that elicited learning (Experiment 2). Although natural-fast speech recognition improved between sessions in Experiment 1, this improvement cannot be attributed to the transfer of learning of time-compressed speech because while learning itself was stronger in the training than in the rapid-learning group, improvements in the recognition of natural-fast speech did not depend on group. Therefore, improvements likely reflect relatively rapid learning of natural-fast speech rather than transfer. Likewise, in Experiment 2, even when rapid learning occurred (in the baseline group), it was not reflected in the recognition of time-compressed speech produced by a new talker, similar to findings on training-induced learning of time-compressed speech^16,17^.

Second, if learning was not stimulus specific, increasing the number of talkers or reducing the number of different sentences in Experiment 2 should not have interfered with learning. Yet these manipulations prevented rapid learning, in line with previous reports on the effects of talker variability^29,62,63^. For example, when listening to speech produced by talkers with atypical /s/ or /sh/ pronunciations, adaptation to the unusual sounds was faster when each speaker was presented on its own than when the two were interleaved^62^. Talker variability during learning is thought to support the transfer of learning by providing listeners with a better sample of the systemic variability in the target speech^7,38^. However, this is not necessarily true for time-compressed speech, in which talker variability was found to slow training-induced learning with no effect on learning transfer^29^. Therefore, we suggest that rapid and training-induced learning are similarly specific or general, and consequently that rapid learning of speech reflects perceptual learning rather than merely procedural or task learning. Similar conclusions were reported for non-verbal auditory and visual learning^15,64,65^. If learning emerges once experience with novel speech has provided sufficient familiarity with the characteristics of the target speech, both brief and prolonged practice could yield specific or general learning, depending on the characteristics of the input. For time-compressed speech, we demonstrated that learning is quite talker specific, as discussed above. On the other hand, learning of noise-vocoded speech seems to generalize more broadly across talkers and stimuli^34,36^.

If more training does not change the nature of learning, what does it do? We speculate that multi-session training could provide further opportunities for learning to stabilize and consolidate without changing the overall nature of learning^17,66,67^. This is consistent with the outcomes of both lab-based^16,68,69^ and rehabilitation-oriented^44,67^ studies. For example, in speech category learning, listeners accumulate information about the acoustic characteristics of the talker over time^68,70^, thus additional experience with a talker is likely to result in additional gains. Gradual accumulation of information about the talkers and the listening context could similarly support learning of perceptually challenging speech beyond the single word level. Furthermore, additional experience gives slower-learning listeners the opportunity to ‘catch-up’. Sadly, most social, educational, and professional environments are not likely to provide those opportunities. Therefore, added to the relative specificity of learning already discussed, it seems that for understanding the role of perceptual learning in speech perception in challenging ‘real-world’ conditions, rapid learning is the key.

### Rapid learning and individual differences in speech perception

The current findings demonstrate that individual differences in rapid between-session perceptual learning are associated with individual differences in the recognition of both natural speech spoken at rapid rates and speech embedded in ‘realistic’ conversational (babble) noise. These findings extend previous works that focused on within-session learning^22,27,28^. The focus on between-session learning made it possible to compare the contributions of rapid and training-induced learning to speech perception on the other tasks (Fig. 5, Table 3). Overall, individuals with good learning were more likely to accurately recognize both natural-fast speech (odds ratio = 1.52) and speech-in-noise (odds ratio = 1.36) than those with poorer learning. Additional training on time-compressed speech by the training group had no significant contribution to speech-in-noise, and a negative contribution to the recognition of natural-fast speech. In the absence of cross-task generalization following training, these findings suggest that in the current study rapid learning makes for the bulk of the speech/learning associations, consistent with the previous findings on rapid within-session learning^22,28^.

The idea that rapid perceptual learning plays an independent role in individual differences in speech perception has merit only to the extent that (rapid) perceptual learning is a general ability or capacity of an individual, at least within a domain. A few recent auditory^25,26^ and visual^23,24^ learning studies suggest that a common factor could explain learning across different tasks. Using visual and auditory discrimination tasks, Yang et al.^24^ reported that despite large differences in learning rates across tasks, a common learning factor accounted for more than 30% of the variance across different learning tasks. Roark et al.^26^ studied the learning of non-speech auditory and visual categories. They found that while learning rates were faster for visual than for auditory categories, categorization accuracy at the end of training was correlated between the auditory and the visual task, suggesting that individual differences in category learning are correlated across the auditory and the visual modalities. As for associations across speech learning tasks, Heffner and Myers^25^ recently found that speech sound learning in different tasks (native and nonnative speech), formed a common factor which they termed phonetic plasticity, which was distinct from a cognitive factor that grouped attention and memory tasks. We note that accuracy data of the type we normally collect might be insufficient to address this issue given the analytical methods used in the studies that reported cross-task association. Furthermore, although the rapid rates of learning in some speech tasks might make it difficult to separate “perception” and “learning”, the replication of the contribution of rapid learning of time-compressed speech to the perception of natural-fast speech and speech-in-noise suggests that this is not an incidental finding. Future studies should nevertheless test the hypothesis that different speech learning conditions cluster around a common factor.

### Limitations & implications

First, sample sizes were not based on a formal power analysis because it was not obvious how to conduct it based on previous data and the rapid rates of time-compressed speech learning. Nevertheless, our previous studies (with similar but not identical conditions) yielded significant group differences as a function of the training protocol with sample sizes of 10 to 24 per group (e.g., ^16,17,29,45^). Current sample size was therefore sufficient to uncover similar or larger effects. Furthermore, for the learning/recognition associations reported here (Table 3), the effect sizes for learning (converted to odd ratios) were similar to those reported for within-session learning^22^ (1.52 vs 1.44–1.68 for natural-fast speech and 1.36 vs. 1.49 for speech-in-noise) with similar groups sizes.

Second, our findings suggest that perceptual learning for speech is largely acoustically specific. However, this is not to say that longer training can never be useful. Instead, training-based studies or interventions should consider the specificity of learning in their design and expected outcomes. Recent work on dysarthric speech suggests that this could be feasible^71^. Learning of dysarthric speech is constrained by the characteristics of individual patients^72^, and even experienced clinicians still benefit from talker-specific training^39^. Therefore, it is proposed that communication partners train to improve the intelligibility of specific patients (e.g., a family member), accounting for learning specificity^71^.

Third, speech perception under challenging conditions incorporates both stimulus (e.g., talker, input distribution;^42^) and listener (e.g., age, language, and cognition^73,74^) related factors. We now suggest that rapid learning is another meaningful listener related factor that could determine how well individual listeners adapt to new or changing auditory environments. Determining if individual differences are associated across different learning tasks or with performance in other situations requires further studies with different learning and perception tasks. Still the finding that individual differences in rapid learning with one type of challenging speech predicts individual differences in the processing of a different type of challenging speech is telling despite the correlational nature of our work.
